# Synthesis of Orthorhombic Tin Dioxide Nanowires in Track Templates

**DOI:** 10.3390/ma17061226

**Published:** 2024-03-07

**Authors:** Zein Baimukhanov, Alma Dauletbekova, Diana Junisbekova, Valeriy Kalytka, Abdirash Akilbekov, Aiman Akylbekova, Guldar Baubekova, Gulnara Aralbayeva, Assyl-Dastan Bazarbek, Abay Usseinov, Anatoli I. Popov

**Affiliations:** 1 Department of Technical Physics, L.N. Gumilyov Eurasian National University, Satpayev Str. 2, Astana 010008, Kazakhstan; zeinb77@mail.ru (Z.B.); diana911115@gmail.com (D.J.); aiman88_88@mail.ru (A.A.); guldar_87@mail.ru (G.B.); agm_555@mail.ru (G.A.); usseinov_ab@enu.kz (A.U.); 2 Faculty of Energy, Automation and Telecommunications, Abylkas Saginov Karaganda Technical University, Karaganda 100027, Kazakhstan; valerii.kalytka@gmail.com; 3 Department of Space Technique and Technology, L.N. Gumilyov Eurasian National University, Satpayev Str. 2, Astana 010008, Kazakhstan; asyl.bazarbek.92@mail.ru; 4 Institute of Solid State Physics, University of Latvia, Kengaraga 8, LV-1063 Riga, Latvia; anatoli.popov@cfi.lu.lv

**Keywords:** track technologies, SiO_2_/Si track template, electrochemical deposition, oxide semiconductors, nanowires, hybrid DFT calculations

## Abstract

Electrochemical deposition into a prepared SiO_2_/Si-p ion track template was used to make orthorhombic SnO_2_ vertical nanowires (NWs) for this study. As a result, a SnO_2_-NWs/SiO_2_/Si nanoheterostructure with an orthorhombic crystal structure of SnO_2_ nanowires was obtained. Photoluminescence excited by light with a wavelength of 240 nm has a low intensity, arising mainly due to defects such as oxygen vacancies and interstitial tin or tin with damaged bonds. The current–voltage characteristic measurement showed that the SnO_2_-NWs/SiO_2_/Si nanoheterostructure made this way has many p-n junctions.

## 1. Introduction

Contemporary materials science is currently focused on developing new materials and methods for oxide photonics, sensors, and optoelectronics [1]. This trend is aimed at creating smaller device sizes, with a particular focus on one-dimensional nanowire-based optoelectronic devices such as emitters [2,3], detectors [4,5], and transistors [6,7]. These devices are currently being actively developed.

SnO_2_ is an oxide semiconductor that is widely recognized for its unique electrical and optical properties. At 300 K, it has a band gap (Eg) of 3.6 eV and exhibits n-type conductivity. Due to its exceptional characteristics, including high electrical conductivity, low electrical resistance, and excellent optical transparency in the visible spectrum, SnO_2_ has been extensively studied for various applications. It is commonly used in the manufacturing of transparent conductors [8], transistors [6,7,9], optoelectronic devices [10,11], gas sensors [12], and more.

There are various types of tin oxide in nanoform. By constructing low-dimensional nanostructures on semiconductor oxides, it is possible to create and design new material systems with unique properties. Nanowires (NWs), for example, can support nanoparticles, other nanowires, and nanosheets, providing access to designs that were previously unattainable with conventional thin-film technology. Molecular beam epitaxy (MBE) and chemical vapor deposition (CVD) are regulated processes used to produce high-quality nanomaterials. The interaction between the physical characteristics of oxides and the 1D shape of NWs makes oxide wide-gap semiconductors (WBGs) an excellent technological foundation.

Producing oxide nanomaterials with consistent morphologies and physical properties is a major challenge due to their lack of repeatability. Unlike group III-V semiconductors, manufacturing such structures is often complex and poorly understood. However, self-assembly mechanisms can provide the necessary repeatability and facilitate the “bottom-up” fabrication method [13].

One of the simplest techniques for creating nanowires is using nanoporous templates. The template synthesis method, which utilizes porous materials such as track membranes made of polyethylene terephthalate (PET), is a potential strategy for creating nanostructures. Electrochemical deposition can produce Fe/Co nanotubes on these PET membranes [14]. Other studies have produced Ni/Fe nanotubes and silver/gold nanoparticle-embedded nanotubes [15,16]. In a separate study, a simple process for electrochemical deposition in PET membranes was proposed to create nanotubes made of zinc. That study found that by annealing the resulting nanotubes, it is possible to control the production of an oxide phase in the nanostructure.

Due to their compatibility with existing silicon technology and their potential for application in the creation of track templates, thin nanoporous SiO_2_ layers incorporated into silicon wafers present intriguing advantages for nanotechnology. These templates, which are made up of nanoporous arrays that have been etched onto the location of latent tracks in SiO_2_, can be filled with a range of substances and composites.

The resultant structures could be used as low-temperature magnetic field sensors [17], biosensors [18,19], active electrical circuit elements [20], and more. These structures on Si wafers were produced using SHI track technology, which is only one illustration of the potential this novel strategy offers.

Due to the self-organization of WBG inside nanochannels, different structures can be obtained using this method. The template was created from a SiO_2_/Si structure using track technology, which includes irradiation with swift heavy ions and a chemical etching process [21,22]. Next, filling the nanopores with various materials is carried out. In our case, we are considering the possibility of tin dioxide precipitation.

An attractive aspect of template synthesis [23] is the ability to tailor a nanomaterial’s physical, chemical, and electronic properties through controlled manipulation of morphology, pore density, shape, and size. Our works demonstrate successful template synthesis of ZnO [23], CdTe [24], and ZnSe_2_O_5_ [25], resulting in stable phases of these compounds as well as phases that are typically only obtainable under special conditions.

This study aimed to form SnO_2_-NWs/SiO_2_/Si nanoheterostructures with arrays of p-n junctions and experimental/theoretical investigations of physical properties of obtained nanostructures. In order to corroborate our experimental results and better understand the electronic structure of the resulting SnO_2_ nanostructures, we simulated the electronic band structure along with the total density of states using the CRYSTAL-17 program [26]. Calculation details are presented in the Materials and Methods section.

## 2. Materials and Methods

In the present work, the SiO_2_/Si (p-type) structure was formed by thermal oxidation of silicon substrate in a wet oxygen atmosphere at T = 900℃. According to ellipsometry, the thickness of the oxide layer was 700 nm. Irradiation of 10 × 10 mm^2^ SiO_2_/Si samples to create latent tracks in the SiO_2_ layer was carried out at a DC-60 cyclotron (Joint Institute for Nuclear Research (JINR) Dubna, Russia). The samples were bombarded at normal incidence with 200 MeV ^132^Xe ions to a fluence of 10^8^ cm^−2^.

Etching in 4% aqueous HF solution was carried out to form nanoporous SiO_2_ layers irradiated with Xe ions. The etchant included m(Pd) = 0.025 g. The process of etching was performed at room temperature for a certain duration. The nanopore sizes were controlled depending on the etching time. After treatment in HF, the samples were washed in deionized water (18.2 MΩ). Electrochemical deposition (ECD) and chemical deposition (CD) were used to fill the nanochannels [27]. The template synthesis was carried out immediately after sensitization of surface and etching. The template synthesis (chemical and electrochemical deposition of materials) was a universal and simple method of receiving arranged arrays of nanostructures in matrix channels.

The electrolyte used to obtain SnO_2_-NWs/SiO_2_/Si, contained 6 g/L SnCl_2_–25 mL H_2_O–2 mL HCl. The composition solution was stirred using a magnetic stirrer while adding hydrochloric acid dropwise until the pH was between 2 and 4, stirring continuously until a clear solution formed. For the ECD process, a cell that was specifically prepared and a VersaStat 3 potentiostat were utilized. The ECD process was carried out at room temperature. A two-beam scanning microscope controlled the filling of nanopores, the Zeiss Crossbeam 540 (Jena, Germany).

X-ray diffraction analysis (XRD) provided detailed information on the structure and phase composition of the samples. Diffractograms were recorded using a Rigaku SmartLab X-ray diffractometer (Rigaku, Tokyo, Japan) with a high-energy resolution 2D HPAD detector HyPix3000 (Rigaku, Tikyo, Japan) in the 2θ range from 5 to 70° at 40 kV. When analyzing the diffraction patterns, we used TOPAS 4.2 software and the international ICDD database (PDF-2 Release 2020 RDB) to identify the phase composition and unit cell parameters of substances. This method enabled us to determine the structures of over 200,000 different compounds.

Photoluminescence spectra were measured at room temperature using a spectrofluorometer CM2203 (Solar, Minsk, Belarus) in the spectral range from 320 to 600 nm when excited by light with a wavelength of λ = 240 nm. Using two double monochromators ensured a minimum level of interference, guaranteeing high measurement accuracy.

A VersaStat 3 potentiostat/galvanostat (Ametek, Berwyn, PA, USA) was used to study the electrical properties of the resulting nanowire arrays. Current–voltage characteristics were measured from an array of filled nanochannels with an area of 0.7 cm^2^.

As noted above, we performed hybrid “large scale” DFT calculations of the structural and electronic properties of obtained SnO_2_ nanostructures in the framework of a periodic linear combination of atomic orbitals (LCAO) approximation. All calculations were made using the primitive crystal cell containing 24 atoms. The all-electron Gaussian-type basis sets (BS) for Sn and O atoms were taken from refs. [28,29], respectively. The total energy convergence threshold for the self-consistent field (SCF) procedure was chosen at 10^7^ Hartree for structure relaxation calculations. The exchange and correlation effects were treated by using a B3LYP functional form (i.e., Becke’s three-parameter hybrid exchange functional [30] and Lee, Yang, Parr correlation functional [31]). It is worth noting that the hybrid B3LYP functional allows us to perform very accurate calculations of the band gap which are in good agreement with the corresponding experimental values. The integration of the reciprocal space was performed with a Pack–Monkhorst 4 × 4 × 4 grid. The effective atomic charges were determined using the Mulliken population analysis [32].

## 3. Results and Discussion

### 3.1. SEM and XRD Analysis of Deposited Samples

**Figure 1:**
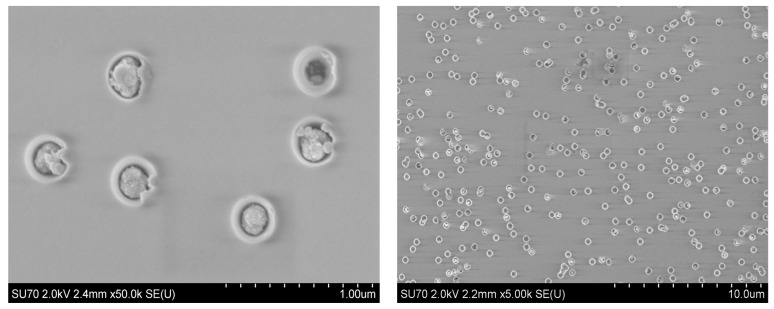
SEM image of the n-type template surface after ECD at 1.75 V for 10 min.

Figure 1 shows SEM images of the surface after deposition.

Figure 1 shows the SEM images of the surface after electrochemical deposition. SEM image analysis revealed nanopore diameters ranging from 519 nm to 562 nm. The amount of filled nanochannels was 87%.

**Figure 2:**
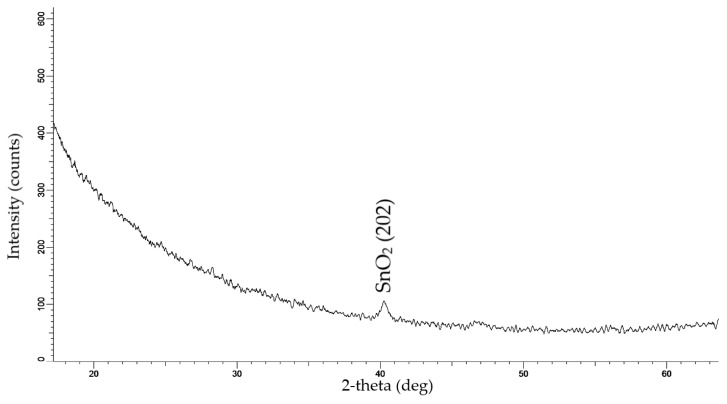
X-ray diffractogram of samples obtained by the ECD for 10 min, at U = 1.75 V.

According to XRD data (Figure 2), electrochemical deposition in a chloride solution into a SiO_2_/Si track template led to the creation of SnO_2_ nanowires with an orthorhombic structure and *Pbca* (61) space group symmetry. The results of the XRD analysis of the sample are summarized in Table 1.

**Figure 3:**
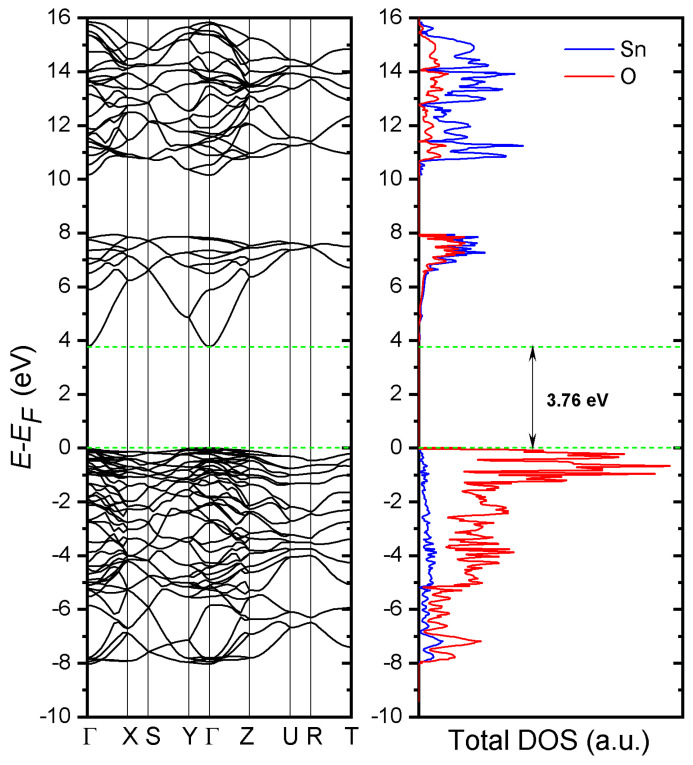
Band structure and total density of states of pure SnO_2_ crystal. The green dotted lines mark the band edges that separate the 3.76 eV bandgap. The Fermi level corresponds to 0 eV.

We calculated the band structure along the highly symmetric k-points of the Brillion zone along with the density of states (Figure 3). The lattice parameters of relaxed crystal geometry were also calculated (see Table 1) The maximum of the valence band and the bottom of the conduction band were located at the Г-point with a band gap of 3.76 eV, which had good agreement with the previous studies using GGA-PBE [33] and the augmented plane wave (APW) methods [34]. It is worth noting, however, that various experimental estimates of the band gap vary from 1.7 to 4 eV [35,36,37]. Nagasawa et al. [38] studied the temperature dependence of the absorption edge for two polarizations of light; they showed a strong dependence of the optical adsorption edge on both factors. For both polarizations, the band gap decreases with increasing temperature. Figure 3 shows good agreement between theory and experiment. In particular, we find a valence band width of ~8 eV in good agreement with both experimental data (7.5 eV reported in ref. [39]) and previous first-principles calculations (7.9 eV and 8.8 eV reported in ref. [40] using PSP and USP, respectively). O-2p states mainly form the uppermost valence band, while the bottom of the conduction band is mostly the result of the contribution of Sn-4d orbitals with a hybridization of O-2p orbitals.

It is known that SnO_2_ crystallizes as a single crystal in the rutile, tetragonal structure (cassiterite) phase SnO_2_-(T). Rutile was typically used as the crystalline phase when this material was created as a nanostructure. However, as with many other materials, the crystal lattice changes under special conditions, such as high pressure, and the crystallographic phase becomes different. The study of [41] was one of the first to synthesize the orthorhombic phase SnO_2_-(O) of tin dioxide. The synthesis process was carried out using a split-sphere high-pressure vessel featuring an inner and outer layer. The container holds the sphere with samples, which is immersed in liquid. As the fluid pressure rises, the sphere is uniformly compressed. The samples in the center of the sphere are subjected to controlled pressure. The temperature was maintained by a small furnace tube. In this experiment, the SnO_2_-(O) polymorph with lattice parameters a = 4.714 Å, b = 5.727 Å, and c = 5.214 Å was synthesized at a pressure of 15.8 GPa and a temperature of 800 °C.

**Figure 4:**
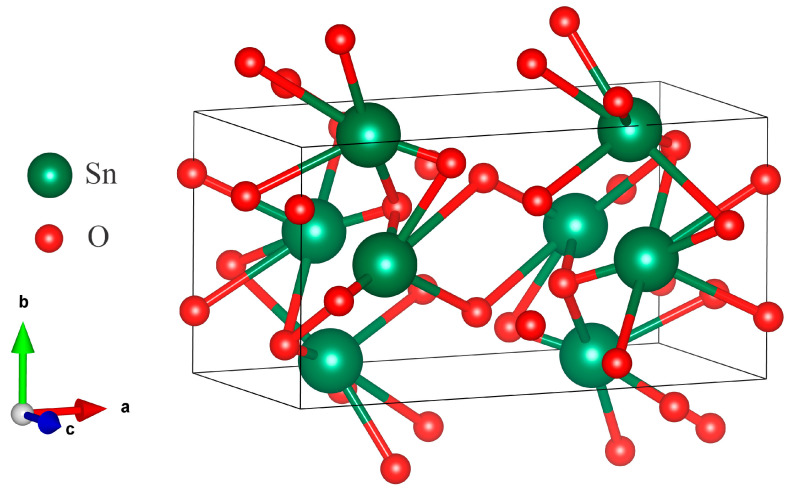
The SnO_2_ polymorph with ZrO_2_-type (Pbca) structure.

Unique studies of polymorphic transformations in SnO_2_ (cassiterite) were conducted in [42]. In situ, XRD analysis of the structure at increasing pressure and temperature showed the existence of four phase transitions up to 117 GPa. Cassiterite powder was mixed with 10 wt% Pt and located into a special cell. Platinum was used as a laser absorber and pressure standard. Starting from the rutile structure, the sequence of polymorphic transformations is as follows: rutile-type with space group *P4_2_*/*mnm* transforms to CaCl_2_−type, *Pnnm*, which then transforms to pyrite-type, *Pa3*. The pyrite-type, *Pa3*, then transforms to ZrO_2_ orthorhombic phase I (O I), *Pbca*, and the last transformation is to cotunnite-type (*Pnam*) orthorhombic phase II (O II). The first three polymorph phases were found to be in general agreement with the results of previous studies. The orthorhombic phase O I and orthorhombic phase O II were observed in SnO_2_ for the first time. So, the (O I) *Pbca* phase formed at room temperature and 50–74 GPa pressure. The lattice parameters for this structure were determined and are as follows: a = 9.304 Å, b = 4.893 Å, and c = 4.731 Å. These values closely resemble those obtained in track template synthesis by ECD and our theoretical calculations (Table 1). Thus, the template synthesis (ECD) yielded orthorhombic SnO_2_ with a ZrO_2_-type crystal structure (orthorhombic phase I). We created a unit cell of SnO_2_ *Pbca* using our own data (Figure 4).

As can be seen, orthorhombic SnO_2_ is more difficult to fabricate as high pressure and temperature are required. But creating orthorhombic SnO_2_ in nanoforms, in the form of thin films, turns out to be a more affordable option. Several research groups have successfully created orthorhombic SnO_2_ thin films using different techniques at moderately low pressures and temperature [43,44,45,46,47,48,49].

Only a small fraction of the studies mention the preparation of the orthorhombic phase of tin dioxide nanowires, although many papers are devoted to the preparation of tin dioxide NWs (see [50,51,52,53] and references cited therein). SnO_2_ nanoribbons/nanowires were synthesized using elevated temperature synthesis techniques in inert Ar gas [54,55]. The authors suggest that orthorhombic SnO_2_ may be the result of product formation in an oxygen-deficient environment. The description of atypical structures in nanowires created using a template method or by adding catalytic in a vapor–liquid–solid method for different materials is presented in [56,57,58]. The authors of [59] conducted a study on the synthesis of pure single-crystal orthorhombic SnO_2_ as well as SnO_2_ nanowires that were decorated with cassiterite nanoclusters.

Based on our literature analysis, we found that using the template synthesis method provides us with the opportunity to successfully obtain tin dioxide with ZrO_2_ orthorhombic phase I (O I), *Pbca* nanowires, and nanoheterostructure (SnO_2_-NWs/SiO_2_/Si).

### 3.2. The Photoluminescence (PL) End Electrical Properties of Orthorhombic SnO_2_-NWs/SiO_2_/Si

Photoluminescence (PL) techniques are useful in detecting nanocrystal structure, defects, and impurities. Previous studies on the luminescence of SnO_2_ nanocrystals can be found in the following articles and references therein [60,61,62,63,64,65,66,67,68,69,70,71,72,73,74,75,76].

**Figure 5:**
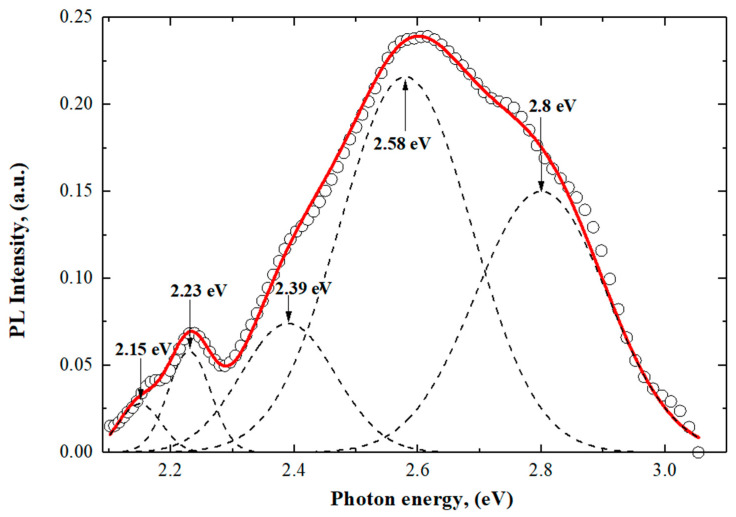
The photoluminescence spectrum of SnO_2_-NWs/SiO_2_/Si structures is decomposed into Gaussian components; SiO_2_ luminescence is taken into account in the PL spectrum.

The PL of SnO_2_-NWs/SiO_2_/Si was investigated in the spectral range from 300 to 600 nm under excitation at λ = 240 nm. In Figure 5, the photoluminescence spectrum of SnO_2_-NWs/SiO_2_/Si structures is represented through Gaussian decomposition. We also subtracted the luminescence of amorphous silica.

**Figure 6:**
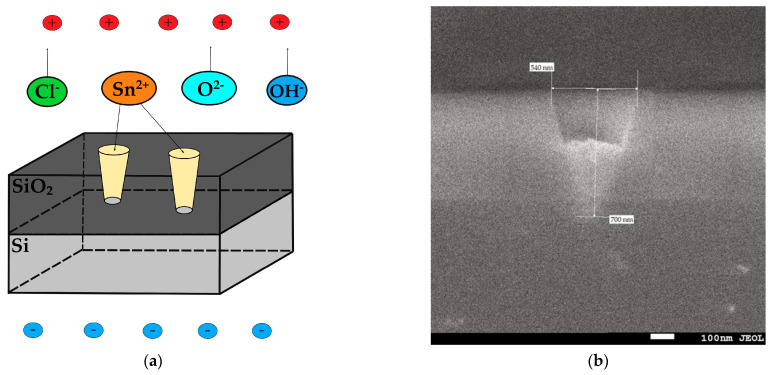
(**a**) Preparation of orthorhombic SnO_2_ nanowires by ECD in SiO_2_/Si and (**b**) the cross-section of filled template.

The photoluminescence is caused by crystal defects or electronic transitions related to oxygen vacancies or interstitial tin, etc. They arise in the band gap during growth. Oxygen vacancies are the most common defects and usually act as emitting centers in luminescence processes. Oxygen vacancies are found in semiconductor oxides in three charge states: $V_{O}^{0}$, $V_{O}^{+}$ and $V_{O}^{2+}$ [70]. $V_{O}^{0}$ is a very shallow donor; it corresponds to a peak of 2.39 eV (518.76 nm) [71], and most oxygen vacancies will be in the paramagnetic state $V_{O}^{+}$ with peak at 2.58 eV (480 nm) [67,72]. The transition from the triplet state to the ground state for $V_{O}^{0}$ may be associated with blue emission at a maximum of 2.8 eV (442.8 nm) [73]. Nanostructured SnO_2_ was found to have a similar observation in its PL spectrum [74]. The luminescence centers responsible for the maximum violet emission at 2.9 eV (427.53 nm) can be attributed to interstitial tin or tin with damaged bonds [65,66,67,68,69]. The peak at 2.15 eV (575 nm) is caused by trap emission from defect levels in the band gap, such as oxygen vacancies, rather than a direct electronic transition. In this SnO_2_-NWs/SiO_2_/Si nanoheterostructure, oxygen vacancies act as luminescent centers, forming defect levels that capture electrons from the valence band and contribute to luminescence [75,76]. It is probable that the observed peak of 2.23 eV (554 nm) is a result of oxygen vacancies that occur during deposition, as reported in studies [77,78]. Similar outcomes were discovered in the examination of SnO_2_ nanobelts [79] and beak-shaped nanorods [80]. It is widely understood that oxygen vacancies are the most frequent type of imperfection and often act as emitting defects in luminescence occurrences. Indeed, from the analysis of the PL spectrum, we can see that the dominant defects are oxygen vacancies, but not defects associated with Sn. This indicates oxygen deficiency. This deficiency can be explained by the electrochemical deposition conditions shown in Figure 6a.

On the inner walls of nanochannels in amorphous silicon dioxide there are silicon and oxygen ions and their vacancies. When an external electric field is applied, the top layer of silicon is charged negatively, and the created field prevents the free movement of oxygen radicals. At the same time, tin ions rush into the channel and interact with oxygen ions on the channel walls (amorphous SiO_2_) to form tin dioxide under conditions of oxygen deficiency, forming orthorhombic tin dioxide with various oxygen-vacancy defects. This also explains the low number of defects associated with tin.

**Figure 7:**
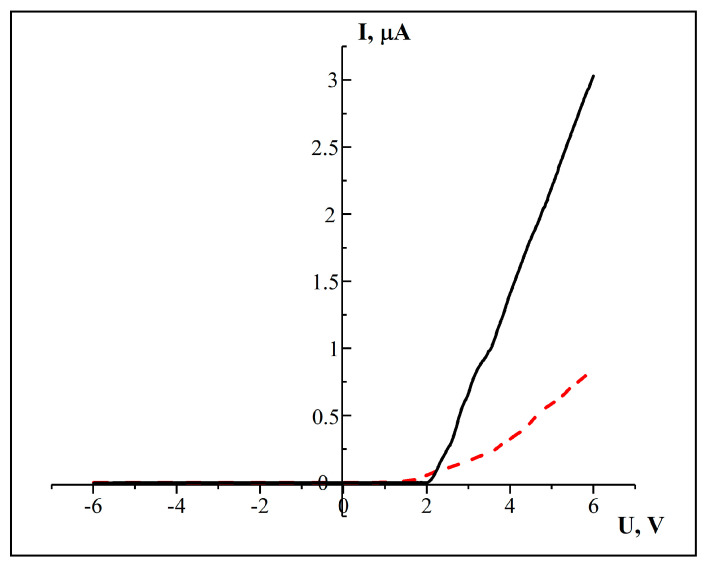
Current–voltage characteristics of SnO_2_-NWs/SiO_2_/Si: dashed curve—initial sample; solid curve—with precipitated SnO_2_ (t_deposition_ = 10 min).

To confirm the contact between SnO_2_ nanowires and silicon substrate, which can be clearly seen in Figure 6b, the current–voltage characteristics (CVCs) of the SnO_2_-NWs/SiO_2_/Si structure were investigated. To confirm the formation of junctions more clearly, a cross-sectional view is shown in Figure 7. It appears that SnO_2_ nanowires are tightly packed onto a silicon substrate and form junction structures. The CVCs were measured from an array of filled nanochannels with an area of 0.7 cm^2^. The CVCs were investigated using a second-order polynomial fitting [25].

Based on Figure 7, it is evident that the CVCs behave like a diode. This means that the current increases exponentially as the voltage increases in the forward direction. The current is attributed to electrons, as the Si substrate is of p-type. By analyzing the CVCs, it can be inferred that the SnO_2_-NWs/SiO_2_/Si structure has an electronic type of conductivity. We can determine the conductivity of nanowire arrays using Formula (1):

(1) $$\sigma =\frac{dI}{dU}\;\frac{l}{A},$$

where l is the length of the nanowire (approximately corresponds to the thickness of the oxide layer of the substrate, about 700 nm); A—area; dI/dU—tangent of the angle of inclination I–U. Values for $A=2\pi r^{2}=57174\;{\mathrm{nm}}^{2}$, $\sigma =1.5\times 10^{8}\;{\mathrm{Om}}^{-1}\cdot {\mathrm{cm}}^{-1}$. Therefore, we can discuss the formation of a series of p-n junctions.

The conductivity of polycrystalline samples can be explained using diffusion and thermoemission models. When the barrier width W is much larger than the free path length of carriers L, we can use the diffusion theory. On the other hand, the thermoelectron emission model is applied when L > W. According to this model, only those carriers whose kinetic energy is greater than the barrier height can cross the boundary. If we assume that we have barriers of the same type, and on average a voltage of *V*/*m* is applied (where m is the number of barriers between the electrodes, and *V* is the interelectrode voltage), then we can use the following equation to determine the height of the potential barrier *φ* and the number of barriers m in series [81]:

(2) $$I=I_{0}\exp\left[-e\left(\varphi -\frac{V}{m}\right)\right]/kT,$$

This equation is also used to analyze current transfer in polycrystalline gallium phosphite [82,83]. The number of barriers can be estimated using the Formula (3):

(3) $$m=\;\frac{H}{h_{k}},$$

where *H* is the height of the nanopore and *h_k_* is the linear size of the nanocrystallite. The average value of the lattice parameters from Table 2 can be used for *h_k_*.

## 4. Conclusions

We successfully obtained vertical nanowires of tin dioxide (SnO_2_) through electrochemical deposition into a SiO_2_/Si track template; they had an orthorhombic ZrO_2_ crystal structure with the lattice parameters a = 9.97195 Å, b = 5.11601 Å, and c = 5.03283 Å.

We calculated the band structure along the highly symmetric k-points of the Brillion zone along with the density of states. The lattice parameters of relaxed crystal geometry were also calculated and matched well with our experimental data. The maximum of the valence band and the bottom of the conduction band were located at the Г-point with a band gap of 3.76 eV, which had good agreement with previous studies.

The study of the PL spectrum showed a broad emission band in the spectral range of 400–600 nm, in which it was found that the dominant defects were oxygen vacancies. Also, maximums were found which were formed by interstitial tin or tin with damaged bonds.

Analysis of the CVCs of SnO_2_-NWs/SiO_2_/Si heterostructures with orthorhombic crystal structure showed that SnO_2_-NWs/SiO2/Si heterostructures with arrays of p-n junctions were synthesized.

Our proposed template synthesis method has several advantages over other methods. Firstly, it does not require lithography. Secondly, it allows for quick optimization of the template synthesis process. Lastly, it has potential applicability to various material systems.

## Figures and Tables

**Table 1:** Crystallographic characteristics of SnO_2_ nanowires in SiO_2_/Si (-p) track template according to XRD results. The calculated parameters are presented in parentheses.

Phase Name	Structure Type	Space Group	(hkl)	2θ	d, Å	L, nm	FWHM	Cell Parameters, Å	Volume, Å^3^	Density, g/cm^3^	Degree of Crystallinity, %	Phase Content, %
SnO_2_	Orthorhombic	Pbca (61)	202	40.219	2.24046	19.39	0.485	a = 9.97195 (10.05) b = 5.11601 (5.10) c = 5.03283 (5.18)	256.76 (266.26)	7.819 (7.57)	41.8	100

**Table 2:** Parameters of intercrystalline barriers in SnO_2_ nanocrystallites.

*H*, nm	*T*, K	*m*	*h_k_*, Å	Barrier Height E, eV
700	300	1044	6.7	3.82 × 10^−2^

## Data Availability

Data are contained within the article.

## References

[1] Lorenz M., Ramachandra Rao M.S., Venkatesan T., Fortunato E., Barquinha P., Branquinho R. (2016). Topical Review: The oxide electronic materials and oxide interfaces roadmap. J. Phys. D Appl. Phys..

[2] Varghese B., Hoong T.C., Yanwu Z., Reddy M.V., Chowdari B.V.R., Wee A.T.S., Vincent T.B.C., Lim C.T., Sow C.H. (2007). Co_3_O_4_ nanostructures with different morphologies and their field-emission properties. Adv. Funct. Mater..

[3] Fang X.S., Yan J., Hu L.F., Liu H., Lee P.S. (2012). Thin SnO_2_ nanowires with uniform diameter as excellent field emitters: A stability of more than 2400 minutes. Adv. Funct. Mater..

[4] Bie Y.Q., Liao Z.M., Zhang H.Z., Li G.R., Ye Y., Zhou Y.B., Xu J., Qin Z.X., Dai L., Yu D.P. (2011). Self-powered, ultrafast, visible-blind UV detection and optical logical operation based on ZnO/GaN nanoscale p-n junctions. Adv. Funct. Mater..

[5] Rigutti L., Tchernycheva M., Bugallo A.D., Jacopin G., Julien F.H., Zagonel L.F., March K., Stephan O., Kociak M., Songmuang R. (2010). Ultraviolet photodetector based on GaN/AlN quantum discs in a single nanowire. Nano Lett..

[6] Tang J.S., Wang C.Y., Xiu F.X., Lang M.R., Chu L.W., Tsai C.J., Chueh Y.L., Chen L.J., Wang K.L. (2011). Oxide-confined formation of germanium nanowire heterostructures for high-performance transistors. Am. Chem. Soc. Nano.

[7] Kulmala T.S., Colli A., Fasoli A., Lombardo A., Haque S., Ferrari A.C. (2011). Self-Aligned coupled nanowire. Am. Chem. Soc. Nano.

[8] Vaseashta A., Dimova-Malinovska D. (2005). Nanostructured and nanoscale devices, sensors and detectors. Sci. Technol. Adv. Mater..

[9] Chou J.C., Wang Y.F. (2002). Preparation and study on the drift and hysteresis properties of the tin oxide gate ISFET by the sol-gel method. Sens. Actuators B Chem..

[10] Lee J.S., Sim S.K., Min B., Cho K., Kim S.W., Kim S. (2004). Structural and optoelectronic properties of SnO_2_ nanowires synthesized from ball-milled SnO_2_ powders. J. Cryst. Growth.

[11] Ying Z., Wan Q., Song Z.T., Feng S.L. (2005). Controlled synthesis of branched SnO_2_ nanowhiskers. Mater. Lett..

[12] Fan Y., Liu J., Lu H. (2012). Hierarchical structure SnO_2_ supported Pt nanoparticles as enhanced electrocatalyst for methanol oxidation. Electrochim. Acta.

[13] Heiss M., Fontana Y., Gustafsson A., Wust G., Magen C., O’Regan D., Luo J., Ketterer B., Conesa-Boj S., Kuhlmann A. (2013). Self-assembled quantum dots in a nanowire system for quantum photonics. Nat. Mater..

[14] Kozlovskii A.L., Kadyrzhanov K.K., Zdorovets M.V. (2018). Structural and Conductive Characteristics of Fe/Co Nanotubes. Russ. J. Electrochem..

[15] Kozlovskiy A., Zhanbotin A., Zdorovets M., Manakova I., Ozernoy A., Kadyrzhanov K., Rusakov V. (2015). Study of Ni/Fe nanotube properties. Nucl. Instrum. Methods Phys. Res. Sect. B Beam Interact. Mater. At..

[16] Mashentseva A., Borgekov D., Kislitsin S., Sdorovets M., Migunova A. (2015). Comparative catalytic activity of PET track-etched membranes with embedded silver and gold nanotubes. Nucl. Instrum. Methods Phys. Res. Sect. B Beam Interact. Mater. At..

[17] Demyanov S., Kaniukov E., Petrov A., Sivakov V. (2014). Positive magnetoresistive effect in Si/SiO_2_ (Cu/Ni) nanostructures. Sens. Actuators A Phys..

[18] Sivakov V., Kaniukov E.Y., Petrov A., Korolik O., Mazmanik A., Bochmann A., Teichert S., Hidi I.J., Schleusener A., Gialla D. (2014). Novel silver nanostructures formation in porous Si/SiO_2_ matrix. J. Cryst. Growth.

[19] Fertig N., Blick R.H., Behrends J.C. (2002). Whole cell patch clamp recording performed on a planar glass chip. Biophys. J..

[20] Hoppe K., Fahrner W.R., Fink D., Dhamodoran S., Petrov A., Chandra A., Saad A., Faupel F., Chakravadhanula V.S.K., Zaporotchenko V. (2008). An ion track based approach to nano-and micro-electronics. Nucl. Instrum. Methods Phys. Res. Sect. B Beam Interact. Mater. At..

[21] Kaniukov E., Bundyukova V., Kutuzau M., Yakimchuk A. (2019). Preculiarities of Formation and Characterization of SiO_2_/Si Ion-Track Template. Fundamental and Applied Nano-Electromagnetics II: THz Circuits, Materials, Devices.

[22] Dallanora A., Marcondes T.L., Bermudez G.G., Fichtner P.F.P., Trautman C., Toulemonde M., Papaleo R.M. (2008). Nanoporous SiO_2_/Si thin layers produced by ion track etching: Dependence on the ion energy and criterion for etchability. J. Appl. Phys..

[23] Giniyatova S., Dauletbekova A., Baimukhanov Z., Vlasukova L., Akilbekov A. (2019). Structure, electrical properties and lum. of ZnO NCs deposited in SiO_2_/Si track templates. Radiat. Meas..

[24] Balakhayeva R., Akilbekov A., Baimukhanov Z., Giniyatova S., Zdorovets M., Gorin Y., Popov A.I., Dauletbekova A. (2020). Structure properties of CdTe nanocrystals created in SiO_2_/Si ion track templates. Surf. Coat. Technol..

[25] Dauletbekova A., Akylbekova A., Sarsekhan G., Usseinov A., Baimukhanov Z., Kozlovskiy A., Vlasukova L.A., Komarov F.F., Popov A.I., Akilbekov A.T. (2022). Ion-track template synthesis and characterization of ZnSeO3 Nanocrystals. Crystals.

[26] Dovesi R., Erba A., Orlando R., Zicovich-Wilson C.M., Civalleri B., Maschio L., Rérat M., Casassa S., Baima J., Salustro S. (2018). Quantum-mechanical condensed matter simulations with CRYSTAL. Wiley Interdiscip. Rev. Comput. Mol. Sci..

[27] Dauletbekova A.K., Alzhanova A.Y., Akilbekov A.T., Mashentseva A.A., Zdorovets M.V., Balabekov K.N. (2016). Synthesis of Si/SiO_2_/ZnO nanoporous materials using chemical and electrochemical deposition techniques. AIP Conference Proceedings.

[28] Laun J., Bredow T. (2022). BSSE-corrected consistent Gaussian basis sets of triple-zeta valence with polarization quality of the fifth period for solid-state calculations. J. Comput. Chem..

[29] Vilela Oliveira D., Peintinger M.F., Laun J., Bredow T. (2019). BSSE-correction scheme for consistent gaussian basis sets of double-and triple-zeta valence with polarization quality for solid-state calculations. J. Comput. Chem..

[30] Becke A.D. (1993). Density-Functional Thermochemistry. III. The Role of Exact Exchange. J. Chem. Phys..

[31] Lee C., Yang W., Parr R.G. (1998). Development of the Colic-Salvetti correlation-energy formula into a Functional of the Electron Density. Phys. Rev. B.

[32] Mulliken R.S. (1955). Electronic population analysis on LCAO–MO molecular wave functions. II. Overlap populations, bond orders, and covalent bond energies. J. Chem. Phys..

[33] Slassi A., Hammi M., Oumekloul Z., Nid-bahami A., Arejdal M., Ziat Y., El Rhazouani O. (2018). Effect of halogens doping on transparent conducting properties of SnO_2_ rutile: An ab initio investigation. Opt. Quantum Electron..

[34] Balakrishnan K., Veerapandy V., Fjellvag V., Vajeeston P. (2022). First-principles exploration into the physical and chemical properties of certain newly identified SnO_2_ polymorphs. ACS Omega.

[35] Arlinghaus F.J. (1974). Energy bands in stannic oxide (SnO_2_). J. Phys. Chem. Solids.

[36] Barbarat P., Matar S.F. (1998). First-principles investigations of the electronic, optical and chemical bonding properties of SnO_2_. Comput. Mater. Sci..

[37] Kucheyev S.O., Baumann T.F., Sterne P.A., Wang Y.M., van Buuren T., Hamza A.V., Terminello L.J., Willey T.M. (2005). Surface electronic states in three-dimensional SnO_2_ nanostructures. Phys. Rev. B.

[38] Nagasawa M., Shionoya S. (1971). Temperature dependence of the fundamental optical absorption edge in stannic oxide. J. Phys. Soc. Jpn..

[39] Themlin J.M., Sporken R., Darville K., Caudano R., Gilles J.M., Johnson R.L. (1990). Resonant-photoemission study of SnO_2_: Cationic origin of the defect band-gap states. Phys. Rev. B.

[40] Maki-Jaskari M.A., Rantala T.T. (2001). Band structure and optical parameters of the SnO_2_ (110) surface. Phys. Rev. B.

[41] Suito K., Kawai N., Masuda Y. (1975). High pressure synthesis of orthorhombic SnO_2_. Mater. Res. Bull..

[42] Shieh S.R., Kubo A., Duffy T.S., Prakapenka V.B., Shen G. (2006). High-pressure phases in SnO_2_ to 117 GPa. Phys. Rev. B.

[43] Chen Z., Lai J.K.L., Shek C.H. (2006). Facile strategy and mechanism for orthorhombic SnO_2_ thin films. Appl. Phys. Lett..

[44] Kaplan L., Ben-Shalom A., Boxman R.L., Goldsmith S., Rosenberg U., Nathan M. (1994). Annealing and Sb-doping of Sn—O films produced by filtered vacuum arc deposition: Structure and electro-optical properties. Thin Solid Film..

[45] Lamelas F.J., Reid S.A. (1999). Thin-film synthesis of the orthorhombic phase of SnO_2_. Phys. Rev. B.

[46] Bae J.Y., Park J., Kim H.Y., Kim H.S., Park J.S. (2015). Facile route to the controlled synthesis of tetragonal and orthorhombic SnO_2_ films by mist chemical vapor deposition. ACS Appl. Mater. Interfaces.

[47] Mueller E. (1984). RHEED-Untersuchungen einer grenzschichtstruktur von SnO_2_ auf quarz. Acta Crystallogr. Sect. B Struct. Sci..

[48] Sangaletti L., Depero L.E., Dieguez A., Marca G., Morante J.R., Romano-Rodriguez A., Sberveglieri G. (1997). Microstructure and morphology of tin dioxide multilayer thin film gas sensors. Sens. Actuators B Chem..

[49] Sberveglieri G., Faglia G., Groppelli S., Nelli P., Taroni A. (1992). A novel PVD technique for the preparation of SnO_2_ thin films as C_2_H_5_OH sensors. Sens. Actuators B Chem..

[50] Masuda Y. (2022). Recent advances in SnO_2_ nanostructure based gas sensors. Sens. Actuators B Chem..

[51] Lu S., Zhang Y., Liu J., Li H., Hu Z., Luo X., Gao N., Zhang B., Jiang J., Zhong A. (2021). Sensitive H_2_ gas sensors based on SnO_2_ nanowires. Sens. Actuators B Chem..

[52] Park H., Kim J., Vivod D., Kim S., Mirzaei A., Zahn D., Park C., Kim S.S., Halik M. (2021). Chemical-recognition-driven selectivity of SnO_2_-nanowire-based gas sensors. Nano Today.

[53] Sharma A., Khosla A., Arya S. (2020). Synthesis of SnO_2_ nanowires as a reusable and flexible electrode for electrochemical detection of riboflavin. Microchem. J..

[54] Dai Z.R., Gole J.L., Stout J.D., Wang Z.L. (2002). Tin oxide nanowires, nanoribbons, and nanotubes. J. Phys. Chem. B.

[55] Dai Z.R., Pan Z.W., Wang Z.L. (2003). Novel nanostructures of functional oxides synthesized by thermal evaporation. Adv. Funct. Mater..

[56] Ihn S.G., Song J.I., Kim T.W., Leem D.S., Lee T., Lee S.G., Koh E.K., Song K. (2007). Morphology-and orientation-controlled gallium arsenide nanowires on silicon substrates. Nano Lett..

[57] Arbiol J., Kalache B., Roca i Cabarrocas P., Morante J.R., Fontcuberta i Morral A. (2007). Influence of Cu as a catalyst on the properties of silicon nanowires synthesized by the vapour–solid–solid mechanism. Nanotechnology.

[58] Dauletbekova A., Vlasukova L., Baimukhanov Z., Akilbekov A., Kozlovskiy A., Giniyatova S., Seitbayev A., Usseinov A., Akylbekova A. (2019). Synthesis of ZnO Nanocrystals in SiO_2_/Si Track Template: Effect of Electrodeposition Parameters on Structure. Phys. Status Solidi B.

[59] Arbiol J., Comini E., Faglia G., Sberveglieri G., Morante J.R. (2008). Orthorhombic Pbcn SnO_2_ nanowires for gas sensing applications. J. Cryst. Growth.

[60] Gu F., Wang S.F., Lu M.K., Zhou G.J., Xu D., Yuan D.R. (2004). Photoluminescence properties of SnO_2_ nanoparticles synthesized by sol−gel method. J. Phys. Chem. B.

[61] Chowdhury P.S., Saha S., Patra A. (2004). Influence of nanoenvironment on luminescence of Eu^3+^ activated SnO_2_ nanocrystals. Solid State Commun..

[62] Faglia G., Baratto C., Sberveglieri G., Zha M., Zappettini A. (2005). Adsorption effects of NO_2_ at ppm level on visible photoluminescence response of SnO_2_ nanobelts. Appl. Phys. Lett..

[63] Maestre D., Cremades A., Piqueras J. (2005). Growth and luminescence properties of micro-and nanotubes in sintered tin oxide. J. Appl. Phys..

[64] Gu F., Wang S.F., Song C.F., Lu M.K., Qi Y.X., Zhou G.J., Xu D., Yuan D.R. (2003). Synthesis and luminescence properties of SnO_2_ nanoparticles. Chem. Phys. Lett..

[65] Munnix S., Schmeits M. (1983). Electronic structure of tin dioxide surfaces. Phys. Rev. B.

[66] Chiodini N., Paleari A., DiMartino D., Spinolo G. (2002). SnO_2_ nanocrystals in SiO_2_: A wide-band-gap quantum-dot system. Appl. Phys. Lett..

[67] Vanheusden K., Warren W.L., Seager C.H., Tallant D.R., Voigt J.A., Gnade B.E. (1996). Mechanisms behind green photoluminescence in ZnO phosphor powders. J. Appl. Phys..

[68] Liu Y., Yang Q., Xu C. (2008). Single-narrow-band red upconversion fluorescence of ZnO nanocrystals codoped with Er and Yb and its achieving mechanism. J. Appl. Phys..

[69] Godinho K.G., Walsh A., Watson G.W. (2009). Energetic and electronic structure analysis of intrinsic defects in SnO_2_. J. Phys. Chem. C.

[70] Zhang W.F., Zhang M.S., Yin Z., Chen Q. (2000). Photoluminescence in anatase titanium dioxide nanocrystals. Appl. Phys. B.

[71] Bhatnagar M., Kaushik V., Kaushal A., Singh M., Mehta B. (2016). Structural and photoluminescence properties of tin oxide and tin oxide: C core–shell and alloy nanoparticles synthesised using gas phase technique. AIP Adv..

[72] Rani S., Roy S., Karar N., Bhatnagar M. (2007). Structure, microstructure and photoluminescence properties of Fe doped SnO_2_ thin films. Solid State Commun..

[73] Her Y.C., Wu J.Y., Lin Y.R., Tsai S.Y. (2006). Low-temperature growth and blue luminescence of SnO_2_ nanoblades. Appl. Phys. Lett..

[74] Hu J.Q., Bando Y., Golberg D. (2003). Self-catalyst growth and optical properties of novel SnO_2_ fishbone-like nanoribbons. Chem. Phys. Lett..

[75] Cai D., Su Y., Chen Y., Jiang J., He Z., Chen L. (2005). Synthesis and photoluminescence properties of novel SnO_2_ asterisk-like nanostructures. Mater. Lett..

[76] Sinha S.K., Bhattacharya R., Ray S.K., Manna I. (2011). Influence of deposition temperature on structure and morphology of nanostructured SnO_2_ films synthesized by pulsed laser deposition. Mater. Lett..

[77] Duan J., Gong J., Huang H., Zhao X., Cheng G., Yu Z., Yang S. (2007). Multiform structures of SnO_2_ nanobelts. Nanotechnology.

[78] Zhang L., Ge S., Zuo Y., Zhang B., Xi L. (2010). Influence of oxygen flow rate on the morphology and magnetism of SnO_2_ nanostructures. J. Phys. Chem. C.

[79] Hu J.Q., Bando Y., Liu Q.L., Golberg D. (2003). Laser-ablation growth and optical properties of wide and long single-crystal SnO_2_ ribbons. Adv. Funct. Mater..

[80] He J.H., Wu T.H., Hsin C.L., Li K.M., Chen L.J., Chueh Y.L., Chou L.J., Wang Z.L. (2006). Beaklike SnO_2_ nanorods with strong photoluminescent and field-emission properties. Small.

[81] Touskova J., Tousek J., Klier E., Kuzel R. (1979). Preparation and basic electrical properties of CdTe thick films. Phys. Status Solidi.

[82] Davis E.A. (2005). States in the gap and defects in amorphous semiconductors. Amorph. Semicond..

[83] Belyaev A.P., Rubets V.P., Nuzhdin M.Y. (2003). Electrical properties of cadmium telluride films synthesized in a thermal field with a temperature gradient. Semiconductors.

